# Serum secreted frizzled-related protein 5 levels differentially decrease in patients with hepatitis B virus-associated chronic infection and hepatocellular carcinoma

**DOI:** 10.3892/ol.2014.2256

**Published:** 2014-06-16

**Authors:** CHUAN PENG, XIAOQIU XIAO, BING KANG, SONG HE, JIBIN LI

**Affiliations:** 1 Department of Nutrition and Food Hygiene, School of Public Health and Management, Chongqing Medical University, Chongqing 400016, P.R. China; 2 Laboratory of Lipid and Glucose Metabolism, The First Affiliated Hospital of Chongqing Medical University, Chongqing 400016, P.R. China; 3 Department of Gastroenterology, The Second Affiliated Hospital of Chongqing Medical University, Chongqing 400010, P.R. China

**Keywords:** hepatitis B virus, secreted frizzled-related protein 5, Wnt signaling, hepatocellular carcinoma, hepatitis B virus DNA

## Abstract

The aim of this study was to investigate the characteristics of serum secreted frizzled-related protein 5 (SFRP5), an inhibitor of Wnt signaling, in hepatitis B virus (HBV)-associated infections and hepatocellular carcinoma (HCC) patients. Serum SFRP5 levels were detected in 147 patients with HBV-associated chronic infection or HCC. Compared with the non-HBV-infected and non-HCC group, the HBV-associated chronic infection and HCC groups exhibited decreased serum SFRP5 levels. A significant inverse correlation between serum SFRP5 levels and HBV DNA levels was identified in the HBV-associated chronic infection and HCC groups. Furthermore, SFRP5 levels differentially decreased in patients with HBV-associated diseases, in a manner which was dependent on liver disease status. Compared with patients exhibiting HBV-associated chronic infection, patients with HCC were found to exhibit lower serum SFRP5 levels. The results of the present study indicated that patients with HBV-associated liver infection and HCC exhibited significantly deceased serum SFRP5 levels, which were found to negatively correlate with HBV DNA levels. Serum SFRP5 levels may present a biomarker for the severity of HBV-associated liver infection, and the risk of HCC initiation and progression.

## Introduction

Hepatocellular carcinoma (HCC) is the sixth most common type of cancer and the third most common cause of cancer-associated mortality worldwide (1,2). It has been estimated that 82% of HCC cases occur in developing countries and 55% occur in China (3). Several causes of HCC have been identified, including hepatitis virus infection, aflatoxin B1 and alcoholic liver cirrhosis, among others. One of most significant risk factors is chronic hepatitis B virus (HBV) infection (4).

Increasing evidence suggests that aberrant expression of the Wnt signaling pathway is involved in the development of HCC (5). By binding to their receptors, termed frizzled (Fzd) proteins, on the cell membrane (6), the Wnt proteins exhibit an important function during animal embryogenesis, growth and development (7,8). Previous studies have shown that the Wnt/β-catenin pathway is involved in the tumorigenesis of several cell types; its involvement has been identified in breast, colorectal, lung and bladder cancer, as well as HCC (9–13).

The secreted Fzd-related proteins (SFRPs) family comprises of five members (SFRP1-SFRP5), which share 30–50% sequence similarity with Fzd proteins. These molecules have been identified as possible negative modulators of the Wnt signaling pathway, which bind directly to Wnt proteins or block their receptors (14).

Recently, it has become clear that aberrant epigenetic modifications of tumor-associated genes are involved in liver tumorigenesis. Of these candidate genes, the *sfrp* family members are increasingly being investigated, with regard to their roles and mechanisms during HCC development. One of the most important observations is that the hypermethylation of *sfrps* has been frequently reported in several types of cancer (15,16). Notably, *sfrp1*, *sfrp2* and *sfrp5* methylation has also been frequently detected in HBV-associated chronic hepatitis and liver cirrhosis (17), which are considered to be pre-neoplastic lesions. Downregulated SFRP5 expression at the mRNA and protein levels was also detected in the tissue of other tumor types (18). However, thus far, few studies have investigated the serum levels of SFRP5 in patients with HBV-associated infections and HCC. In the present study, patient characteristics and serum SPFR5 levels were investigated in patients with different HBV-associated liver disease statuses.

## Materials and methods

### Patients

A total of 147 patients with HBV-associated chronic infection or HCC were enrolled at the Second Affiliated Hospital of Chongqing Medical University (Chongqing, China) between March 2013 and July 2013. The patients were divided into two groups based on clinical diagnosis, an HBV group (75 patients with HBV-positive chronic hepatitis or liver cirrhosis) and an HBV-C group (72 patients with HBV-associated HCC). A comparison group (CT group) of 38 subjects without any history of chronic hepatitis, liver cirrhosis or HCC was enrolled at the same hospital. The study was approved by the Ethical Committee of Chongqing Medical University (Chongqing, China) and written informed consent was obtained from all subjects.

### Clinical characteristics of patients

The clinical patient information included age, gender, liver cirrhosis status and serum HBV DNA quantitative analysis results, including serum levels of alanine aminotransferase (ALT), aspartate aminotransferase (AST), albumin, total bilirubin (TB), combining bilirubin (CB) and unconjugated bilirubin (UCB); prothrombin time (PT) and prothrombin activity (PTA). In addition, serum samples from the CT group were collected as normal controls for SFRP5 level comparison.

### Serum SFRP5 level determination

Blood samples were collected from the subjects following overnight fasting. Serum samples were obtained by centrifugation at 4°C and stored at −80°C for future use. Serum SFRP5 levels were determined using the ELISA method (Human ELISA kit; Uscn Life Science Inc., Wuhan, China).

### Statistical analysis

The data are presented as the mean ± standard deviation. Data with a skewed distribution were logarithmically transformed for further statistical analysis. Student’s t-test and analysis of variance were used for serum SFRP5 level comparison. Correlational analysis of serum SFRP5 levels with liver disease status (control, HBV-associated chronic hepatitis, liver cirrhosis and HCC) was performed using Spearman’s rank correlation. All tests were two-tailed and P<0.05 was considered to indicate a statistically significant difference. All statistical analyses were performed using SPSS, version 17.0 (SPSS, Inc., Chicago, IL, USA).

## Results

### Patient characteristics

The clinical characteristics of the patients are shown in Table I. No significant differences were identified in albumin (35.13±8.51 g/l vs. 36.43±8.26 g/l), ALT (156.46±183.20 U/l vs. 95.60±220.02 U/l) or AST (129.04±146.69 U/l vs. 104.15±213.76 U/l) levels between the HBV and HBV-C groups. The mean age of the HBV-C group (53.31±11.93 years) was higher than that of the HBV group (42.37±15.28 years) (P<0.001; Table I). In addition, the mean PT was higher in the HBV group (16.34±3.73 sec) than that of the HBV-C group (14.95±2.19 sec) (P<0.01; Table I). By contrast, the PTA of the HBV group (73.51±23.24) was lower when compared with the HBV-C group (80.89±19.27%) (P<0.05; Table I). In the HBV group, the levels of TB (75.58±109.28 μmol/l vs. 27.87±28.70 μmol/l), CB (50.15±77.26 μmol/l vs. 14.58±17.77 μmol/l), UCB (22.17±34.89 μmol/l vs. 13.29±12.73 μmol/l) and CB/TB (0.59±0.16 vs. 0.50±0.09) were significantly increased (P<0.01; Table I).

### Serum SFRP5 levels decrease in HBV-associated liver diseases

**Figure 1:**
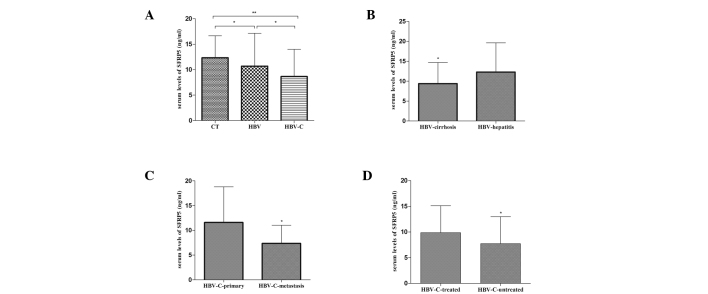
Serum SFRP5 levels in the different subgroups. (A) Comparison of SFRP5 levels in CT, HBV and HBV-C groups (^**^P<0.01, vs. CT group; ^*^P<0.05, vs. HBV group). (B) Comparison of SFRP5 levels in HBV-cirrhosis and HBV-hepatitis subgroups (^*^P<0.05). (C) Comparison of SFRP5 levels in HBV-C-primary and HBV-C-metastasis subgroups (^*^P<0.05). (D) Comparison of SFRP5 levels in HBV-C-treated and HBV-C-untreated subgroups (^*^P<0.05). Error bars represent SD. SFRP5, secreted frizzled-protein 5; CT, control group; HBV, hepatitis B virus; HCC, hepatocellular carcinoma; HBV-C, HBV-associated HCC; HBV-cirrhosis, subgroup of HBV-associated liver cirrhosis; HBV-hepatitis, subgroup of HBV-associated chronic hepatitis without liver cirrhosis; HBV-C-primary, subgroup of HCC patients without metastasis; HBV-C-metastasis, subgroup of HCC patients with metastasis; HBV-C-treated, subgroup of HCC patients receiving medical treatment; HBV-C-untreated, subgroup of HCC patients without medical treatment.

Serum levels of SFRP5 decreased in the HBV (10.69±6.44 ng/ml) and HBV-C (8.65±5.33 ng/ml) groups when compared with the CT group (12.33±4.32 ng/ml)(P<0.05). Notably, the serum SFRP5 levels were decreased in the HBV-C group when compared with the HBV group (P<0.05; Fig. 1A).

### Inverse correlation between serum SFRP5 levels and HBV DNA level

**Figure 2:**
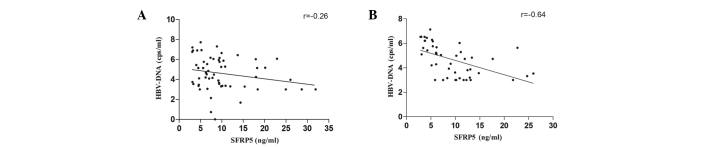
Correlation between serum SFRP5 levels and HBV DNA levels. (A) HBV group (P<0.05) and (B) HBV-C group (P<0.01). HBV DNA levels were logarithmically transformed prior to statistical analysis. SFRP5, secreted frizzled-protein 5; HBV, hepatitis B; HBV-C, HBV-associated hepatocellular carcinoma.

Spearman’s rank correlational analysis identified a significant inverse correlation between serum SFRP5 levels and HBV DNA levels in the HBV (r=−0.26; P<0.05) and HBV-C (r=−0.64; P<0.01) groups (Fig. 2A and B, respectively).

### Differential decrease in serum SFRP5 levels in HBV-associated liver diseases

SFRP5 levels in patients with different liver disease statuses were investigated. Compared with HBV-associated chronic hepatitis patients (12.28±7.34 ng/ml), patients with liver cirrhosis exhibited lower serum SFRP5 levels (9.38±5.31 ng/ml) (P<0.05; Fig. 1B). In the HBV-C group, patients with metastasis exhibited significantly lower SFRP5 levels (7.36±3.67 ng/ml) when compared with those without metastasis (11.58±7.20 ng/ml) (P<0.05; Fig. 1C). Similarly, the SFRP5 levels were significantly lower in patients prior to receiving medical treatment (7.72±5.27 and 9.88±5.25 ng/ml, respectively) (P<0.05; Fig. 1D).

## Discussion

HBV infection is a major public health challenge worldwide. It is estimated that ~30% of the global population exhibit serological evidence of current or past HBV infection (19). HBV infection may cause liver diseases, including acute and chronic hepatitis and liver cirrhosis. HBV infection is also considered to be a major risk factor for the development of HCC. With regard to the process of HBV inducing HCC, numerous observations from previous studies have revealed that abnormal activation of the Wnt pathway is important (20,21). The Wnt proteins are a large family of palmitoylated secreted glycoproteins, which has 19 known members in humans. These secreted proteins bind to the Fzd proteins and low density lipoprotein receptor-associated proteins on the cell membrane (6). Wnt proteins activate at least three different signaling pathways: The Wnt/β-catenin, Wnt/non-canonical and Wnt/Ca^2+^ pathways (22). There are several known types of Wnt pathway antagonists, including the Cerberus, Wnt inhibitory factor 1, SFRP and Dickopf families. The SFRP family comprises five glycoproteins, identified as putative inhibitors of the Wnt signaling pathway. Numerous studies have identified frequent epigenetic inactivation of *sfrp* genes in numerous types of cancer (23,24). However, few studies have investigated the characteristics of SFRP5 levels in serum. In the present study, decreased serum SFRP5 levels were observed in HBV chronic infection and HCC patients when compared with the control group. These results further support the theory that inactivation of the *sfrp5* gene is frequently found in HBV-associated liver diseases. Two different mechanisms are involved in the downregulation of SFRP expression in cancer cells: Allelic loss and epigenetic silencing. SFRP5, together with SFRP1 and SFRP2, has dense CpG islands which flank the first exon. The frequent occurrence of the downregulation of SFRP expression has been observed in several types of cancer due to promoter hypermethylation of these genes (25–27). SFRP1 promoter hypermethylation is a common event occurring in HBV-induced chronic infections and HCC (28). The results of the present study revealed that serum SFRP5 levels were found to negatively correlate with HBV DNA levels. This observation suggested that HBV-associated infection was possibly responsible for decreased SFRP5 serum levels. Furthermore, HBV infection-associated oxidative stress and the HBV encoded X antigen are considered to cause SFRP promoter hypermethylation (29–31).

Another important observation was that serum SFRP5 levels decreased differentially in HBV chronic infection and HBV-associated HCC. SFRP5 levels were found to be lower in HCC patients than in patients with only chronic hepatitis or HBV-associated liver cirrhosis. These results are consistent with previous studies, which revealed that methylation was detected to be weaker in hepatitis and cirrhosis patients when compared with liver cancer cell lines or in primary HCC (17). Therefore, it is possible that low levels of *sfrp* gene promoter methylation or the relatively low serum SFRP5 levels observed in HBV-associated chronic hepatitis and liver cirrhosis may serve as biomarker for the risk of developing HCC.

The results of the current study also indicate that different serum SFRP5 levels may be exhibited among HCC patients according to disease status. Firstly, it was found that patients usually exhibited lower SFRP5 levels prior to receiving any therapy. This observation suggests that therapeutic measurement, including tumorectomy, chemotherapy or antiviral therapy, may contribute to the alleviation of HBV-associated oxidative stress and increase serum SFRP5 levels. However, the association between serum SFRP5 levels and anticancer therapy remains unclear and further investigation is required. Secondly, significantly lower levels of serum SFRP5 were observed in HCC patients who exhibited metastasis. The reason for this is not clear; however, it may involve the expression of other Wnt target genes. Furthermore, Zhao *et al* (32) identified an inverse correlation between SFRP5 expression and matrix metalloproteinase 7 (MMP-7) and membrane type 1-MMP expression, which are known as regulators of invasion and metastasis. Therefore, it is possible that the downregulation of SFRP5 leads to the activation of Wnt pathways and subsequently increases the transcription of Wnt target genes, including *c-myc*, *cyclin D1* and certain *MMPs*. It would be of interest to investigate the association between SFRP5 and these members of the downstream Wnt pathway. This may lead to the development of novel approaches to prevent HBV-associated HCC, as well as novel therapeutic strategies.

Recent studies have investigated the role of *sfrp* gene methylation in the initiation and progression of HCC. However, the majority of these studies have not investigated the characteristics of serum SFRP5 levels. The most significant result obtained from this study is that serum SFRP5 levels differentially decreased in HBV-associated chronic infection and HCC patients. This difference is dependent on the disease condition. These results suggested that serum SFRP5 levels may present a possible biomarker for the severity of HBV-associated chronic infection and the risk of HCC initiation and progression. However, prior to the use of serum SFRP5 as a reliable biomarker in clinical practice, large-scale studies are required to clarify the relevant factors. In addition, exploration of the manner in which the HBV infection triggers epigenetic modulation and subsequently regulates the serum SFRP5 level may aid in understanding the function of SFRP5 in the Wnt signaling pathways.

## Figures and Tables

**Table I:** Clinical characteristics of patients.

Parameter	HBV-associated chronic hepatitis group	HBV-associated HCC group
Total no.	75	72
Males, n	58	64
Females, n	17	8
Age (years ± SD)	42.37±15.28	53.31±11.93^b^
PT (sec ± SD)	16.34±3.73	14.95±2.19^b^
PTA (% ± SD)	73.51±23.24	80.89±19.27^a^
Albumin (g/l ± SD)	35.13±8.51	36.43±8.26
ALT (U/l ± SD)	156.46±183.20	95.60±220.02
AST (U/l ± SD)	129.04±146.69	104.15±213.76
TB (μmol/l ± SD)	75.58±109.28	27.87±28.70^b^
CB (μmol/l ± SD)	50.15±77.26	14.58±17.77^b^
UCB (μmol/l ± SD)	22.17±34.89	13.29±12.73^a^
CB/TB (±SD)	0.59±0.16	0.50±0.09^b^

a P<0.05 and

b P<0.01, compared with the HBV-associated chronic hepatitis group. Data are presented as the mean ± SD.

HBV, hepatitis B virus; HCC, hepatocellular carcinoma; PT, prothrombin time; PTA, prothrombin activity; ALT, alanine aminotransferase; AST, aspartate aminotransferase; TB, total bilirubin; CB, combined bilirubin; UCB, unconjugated bilirubin.
